# Corrigendum: Population dynamics analysis of the interaction between tacrolimus and voriconazole in renal transplant recipients

**DOI:** 10.3389/fphar.2025.1584520

**Published:** 2025-03-25

**Authors:** Zhi-Hua Sun, Yi-Chang Zhao, Jia-Kai Li, Fenghua Peng, Feng Yu, Bi-Kui Zhang, Miao Yan

**Affiliations:** ^1^ Department of Pharmacy, The Second Xiangya Hospital, Central South University, Changsha, Hunan, China; ^2^ School of Basic Medicine and Clinical Pharmacy, China Pharmaceutical University, Nanjing, Jiangsu, China; ^3^ Department of Urological Organ Transplantation, The Second Xiangya Hospital, Central South University, Changsha, Hunan, China; ^4^ International Research Center for Precision Medicine, Transformative Technology and Software Services, Changsha, Hunan, China

**Keywords:** tacrolimus, population pharmacokinetics, voriconazole, renal transplantation, predictive model

In the published article, there was an error in **Affiliation 1**. Instead of “International Research Center for Precision Medicine, Transformative Technology and Software Services, Changsha, Hunan, China”, it should be “Department of Pharmacy, The Second Xiangya Hospital, Central South University, Changsha, Hunan, China.”

There was an error in **Affiliation 2**. Instead of “Basic Medicine and Clinical Pharmacy, China Pharmaceutical University, Nanjing, Jangsu, China”, it should be “School of Basic Medicine and Clinical Pharmacy, China Pharmaceutical University, Nanjing, Jiangsu, China.”

There was an error in **Affiliation 3**. Instead of “The Second Xiangya Hospital, Central South University, Changsha, Hunan, China”, it should be “Department of Urological Organ Transplantation, The Second Xiangya Hospital, Central South University, Changsha, Hunan, China.”

There was an error in **Affiliation 4**. Instead of “Department of Pharmacy, The Second Xiangya Hospital, Central South University, Changsha, Hunan, China”, it should be “^4^ International Research Center for Precision Medicine, Transformative Technology and Software Services, Changsha, Hunan, China.”

The authors apologize for this error and state that this does not change the scientific conclusions of the article in any way. The original article has been updated.

