# Supplementary material for: Population dynamics analysis of the interaction between tacrolimus and voriconazole in renal transplant recipients
Source: Front Pharmacol. 2025 Jan 29;15:1502097. doi: 10.3389/fphar.2024.1502097 (PMC11813913; doi:10.3389/fphar.2024.1502097)
Supplement: Supplementary file 1 [file Table1.DOCX]

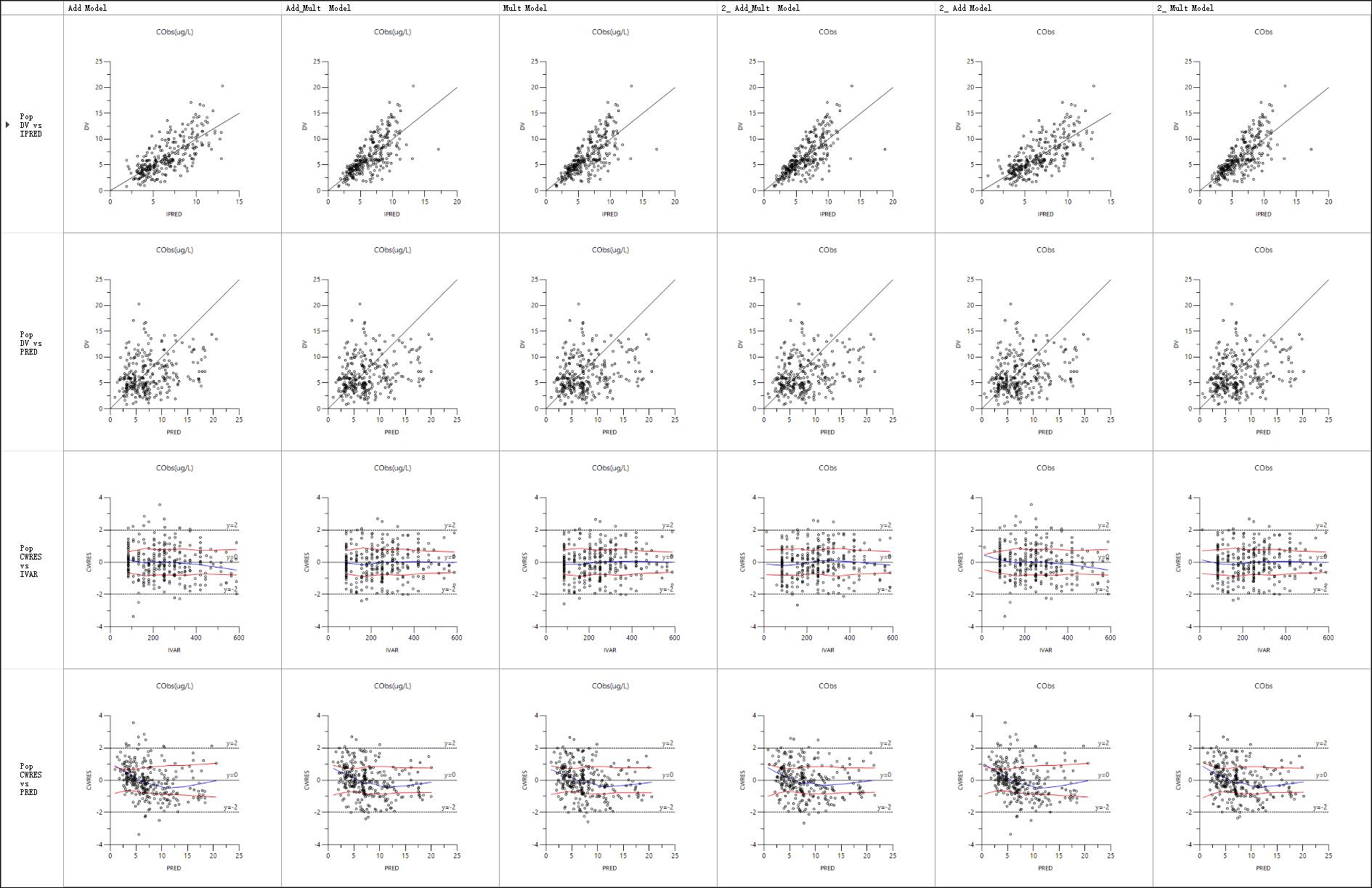


Figure A Basic model fitting diagram

Table A Patient Clinical and Laboratory Parameters

| **Abbreviation** | **Full name** |
| --- | --- |
| C_VRC_ | Voriconazole blood concentration |
| WBC | White Blood Cell |
| RBC | Red Blood Cell |
| NEUT | Neutrophil |
| LYMPH | Lymphocyte |
| PLT | Platelet |
| HGB | Hemoglobin |
| HCT | Hematocrit |
| PCT | Procalcitonin |
| TP | Total Protein |
| ALB | Albumin |
| TBIL | Total Bilirubin |
| DBIL | Direct Bilirubin |
| TBA | Total Bile Acid |
| AST | Aspartate Aminotransferase |
| ALT | Alanine Aminotransferase |
| BUN | Blood Urea Nitrogen |
| CREA | Creatinine |
| Sex | Patient's gender |
| Age | Patient's age |
| Wt | Patient's weight |
