# Supplementary material for: Population dynamics analysis of the interaction between tacrolimus and voriconazole in renal transplant recipients
Source: Front Pharmacol. 2025 Jan 29;15:1502097. doi: 10.3389/fphar.2024.1502097 (PMC11813913; doi:10.3389/fphar.2024.1502097)
Supplement: Supplementary file 2 [file Table2.DOCX]

**Population Dynamics Analysis of the Interaction Between Tacrolimus and Voriconazole in Renal Transplant Recipients**

Zhi-Hua Sun^c,d^ ,Yi-Chang Zhao^a,b,c^, Jia-Kai Li ^a,b,c^, Huai-yuan Liu^c,d^, Hua-Lin Cai^a,b,c^, Fenghua Peng^a^, Feng Yu^d^, Bi-Kui Zhang^a,b,c*^, and Miao Yan^a,b,c*^.

^a^The Second Xiangya Hospital, Central South University, Changsha, Hunan, P.R. China 410011.

^b^Department of Pharmacy, the Second Xiangya Hospital, Central South University, Changsha, Hunan, P.R. China 410011.

^c^International Research Center for Precision Medicine, Transformative Technology and Software Services, Hunan, China.

^d^China Pharmaceutical University, Nanjing, Jiangsu, P.R. China 210009.

^e^Department of Medical Laboratory, the Second Xiangya Hospital, Central South University, Changsha, Hunan, P.R. China 410011.

* **Correspondence:** Bi-Kui Zhang and Miao Yan

**Address:** Department of Clinical Pharmacy, the Second Xiangya Hospital of Central South University, Changsha 410010, Hunan Province, China.

**E-mail:** 505995@csu.edu.cn and yanmiao@csu.edu.cn

**Tel:** 086-0731-85292098

**Abstract:**

**Background:** The concurrent administration of tacrolimus and voriconazole in kidney transplant recipients can lead to drug interactions, potentially resulting in severe adverse reactions. This study aimed to establish a robust population pharmacokinetic model to explore the interaction between tacrolimus and voriconazole in greater depth.

**Methods:** Tacrolimus blood samples and laboratory data were prospectively collected from eligible patients enrolled between April 2023 and April 2024, following predefined inclusion and exclusion criteria. Using Phoenix (version 8.1), a pharmacokinetic prediction model was developed. Model performance was assessed using model fitting plots, bootstrap analysis, and visual predictive checks (VPC).

**Results:** This study ultimately included 51 eligible patients, with a total of 281 blood samples collected. Analysis revealed a significant negative correlation between voriconazole concentration (Cvrc) and tacrolimus volume of clearance rate (CL), a significant positive correlation between platelets (PLT) and tacrolimus clearance (CL), and a significant negative correlation between blood cells (RBC) and tacrolimus clearance (CL).

**Conclusion:** This study successfully established a population pharmacokinetic model for renal transplant patients concurrently receiving tacrolimus and voriconazole. The model demonstrated good predictive performance and offers valuable insights to clinicians for optimizing tacrolimus dosing in this patient population.

**Keywords:** tacrolimus, population pharmacokinetics, voriconazole, renal transplantation, predictive model.

**1. Introduction**

Kidney transplantation significantly improves survival rates for patients with kidney disease^1, 2^. However, effective management is crucial, particularly in immunotherapy and infection control. Voriconazole is commonly used in combination with tacrolimus to treat invasive fungal infections, but this can lead to pharmacokinetic (PK) drug interactions and adverse effects^3, 4^. Because Tacrolimus is mainly metabolized by CYP3A4 and CYP3A5^5-7^, while voriconazole is mainly metabolized by CYP3A4 and CYP2C19. Voriconazole is also a strong inhibitor of CYP3A4/5^8, 9^. The Vfend instruction book recommended a decreased initial dose of tacrolimus during voriconazole co-therapy. The results varied widely and were not suitable for clinical use. Nuria et al^10^ found that PPK-based Tac dosing had significant advantages over classic labeled dosing based on body weight when initiating Tac prescription, However, most studies only analyze voriconazole as an influencing factor, which can only prove that the use of voriconazole is a key influencing factor, which has great limitations^11-13^. In another study, Fay S et al^14^, reviewed 8 transplant recipients (5 lung, 2 redo lung, 1 heart) treated concurrently with flucloxacillin, voriconazole, and tacrolimus. A significant three-way interaction was found between flucloxacillin, voriconazole, and tacrolimus, but no further insight was gained regarding the effects of voriconazole. In addition, A previous retrospective study suggested that voriconazole concentrations could be used to optimize tacrolimus dosing in lung transplant recipients, offering an important perspective^13^. However, this study was limited by its exclusive reliance on trough concentration measurements and the inherent constraints of retrospective data analysis. Notably, there remains a lack of research focusing on renal transplant patients. To address this gap, our study employed a prospective data collection approach combined with a sparse sampling design, allowing us to randomly capture blood concentration data that thoroughly represented both absorption and elimination phases. This methodology enabled a robust and high-resolution pharmacokinetic analysis, providing a more reliable foundation for optimizing voriconazole therapy in renal transplant patients.

**2. Methods**

**2.1 Study subjects**

Study participants consisted of renal transplant patients admitted to the Second Xiangya Hospital of Central South University between April 2023 and September 2024. This research received approval from the hospital ethics committee [LYEC2024-K0106]. This is a non-interventional clinical study, all patients signed informed consent forms, and patient information is strictly confidential.

**2.2 Inclusion and exclusion criteria**

The study employed the following inclusion criteria: (a) Patients undergoing kidney transplantation at the Renal Transplantation Department of Xiangya Second Hospital; (b)Patients aged 18 years or older; (c) Patients administered a triple immunosuppressive regimen comprising tacrolimus, mycophenolate mofetil, and glucocorticoids, alongside oral voriconazole; (d) At least five tacrolimus concentration values were collected for each patient, etc. (e) Patients at least one-year post-kidney transplantation. The exclusion criteria were as follows: (a) Patients concurrently prescribed cyclosporine, rapamycin, or other immunosuppressants; (b) Patients receiving rifampicin, isoniazid, phenytoin, or other potent CYP450 inducers or inhibitors; (c) Patients with incomplete or missing pertinent experimental data, etc.

**2.3 Date collection and analysis**

**2.3.1 Blood Sample Collection and Monitoring for Tacrolimus and Voriconazole**

In this study, both drugs were administered orally. Tacrolimus was provided in an immediate-release (IR) formulation, typically dosed twice daily. The doses of both tacrolimus and voriconazole were adjusted by clinicians based on therapeutic drug monitoring (TDM), established clinical guidelines, and patient-specific factors.

A total of 51 patients were enrolled in the study and randomly assigned to three groups using a sparse sampling design. Each group followed a predefined sampling schedule: Group 1 collected samples at 0, 0.5, and 1 hour; Group 2 collected samples at 2, 4, and 6 hours; and Group 3 collected samples at 8 hours and 30 minutes before the next dose.

Tacrolimus blood concentration was measured using a chemiluminescent particle immunoassay with the ARCHITECT Tacrolimus Kit IL77-35. Detailed information on standard operating procedures, assay methodology, and stability data is provided in the Prograf assay kit instructions (IL77-G08363R10-B1L77C) ^15^.

Voriconazole plasma concentration was determined through a fully automated two-dimensional liquid chromatography system (2D-HPLC, Changsha Demeter Instrument Co., Ltd.). Chromatographic conditions included: Column A (FRO C18, 5 μm, 100 mm × 3.0 mm, ANAX) with a mobile phase of 20 mmol/L ammonium acetate-acetonitrile (48:52, V/V) at a flow rate of 1.0 mL/min, and Column B (ASTON HD C18, 150 mm × 4.6 mm, 5 μm, ANAX) with a mobile phase of 40 mmol/L ammonium acetate-acetonitrile (85:15, V/V) at a flow rate of 1.2 mL/min. The detection wavelength was set at 273 nm, the column temperature maintained at 45°C, and the injection volume set to 200 μL. The method exhibited a linear range of 0.35–11.26 μg/mL. All laboratories underwent annual quality assessments conducted by the National Health Commission Clinical Testing Center to ensure compliance with quality standards.

**2.3.2Pharmacokinetic Data Analysis and Model Evaluation**

Laboratory data including sex, weight, age, albumin, hematocrit, creatinine, aspartate aminotransferase, C-reactive protein, and total bilirubin were collected in this study.Data analysis was performed using Phoenix NLME pharmacokinetic software (version 8.1). For the description of baseline characteristics, mean and standard deviation were used to describe normally distributed continuous variables, median and interquartile range were used to describe non-normally distributed continuous variables, and frequency and percentage were used for categorical variables.

In the process of establishing the structural model, both one-compartment and two-compartment models were evaluated. Key parameters such as LogLik, AIC, OFV, and Shrinkage were compared between the models, alongside the analysis of the fitting diagram (Figure A). Based on these comparisons, the one-compartment Add-Multiplicative model for oral absorption and elimination was selected as the most suitable. The final covariate model was selected by a stepwise method based on the least squares principle, including forward inclusion (p<0.05) and backward elimination (p<0.01). The change in the model objective function value (ΔOFV) after the inclusion of covariates was evaluated. The final model was evaluated using methods such as goodness-of-fit plots, bootstrap analysis, and visual predictive tests (VPC). The research flow chart is shown in Figure 1.

**3. Results**

**3.1 Patient demographics and characteristics**

A total of 51 patients were ultimately enrolled in this study, comprising 38 males. The study gathered a total of 281 tacrolimus concentration points, with a median concentration of 5.8 ng/ml. Detailed demographic data can be found in Table 1

Table 1 demographics and characteristics

| Parameter | Value |
| --- | --- |
| Total number of patients | 51 |
| Male, n (%) | 37 (72.55) |
| Female n (%) | 14 (27.45) |
| Age | 45.00[38.00, 55.00] |
| Wt (kg) | 59.60 [51.10,67.20] |
| Ctac (ng/ml) | 5.80 [4.20,8.65] |
| Cvrc (ug/ml) | 0.95 [0.00,2.64] |
| WBC (10^9^/L) | 6.10 [4.48,8.62] |
| RBC (10^12^/L) | 3.44 [2.82,4.12] |
| LYMPH (%) | 9.8 [5.60,19.40] |
| NEUT (%) | 82.10 [70.30,88.20] |
| HCT (%) | 31.80 [25.10, 37.10] |
| HGB (g/L) | 102.00 [78.00, 118.00] |
| PLT (10^9^/L) | 170.00 [129.00,220.00] |
| ALT (U/L) | 12.60 [8.30, 20.30] |
| AST (U/L) | 18.20 [14.00, 24.40] |
| TBIL (μmol/L) | 5.40 [4.10, 7.70] |
| DBIL (μmol/L) | 2.40 [1.70, 3.30] |
| TBA (μmol/L) | 3.40 [2.00, 5.90] |
| ALB(g/L) | 34.40 [30.65,37.70] |
| BUN (mmol/L) | 15.80 [10.00, 23.40] |
| CREA(μmol/L) | 172.00 [120.00, 277.00] |
| TP (g/L) | 53.10 [48.50, 58.40] |
| PCT (%) | 0.17 [0.13, 0.22] |

Measurement data are presented as median (interquartile range) and categorical data were expressed as frequencies.

**3.2 Establishment of population pharmacokinetic model**

After thorough consideration of various models encompassing one-compartment/two-compartment additive, multiplicative, and mixture of oral absorption and elimination, the Add-Multiplicative model emerged as the chosen basic model, with AIC=1411.82, BIC=1433.65 (Table 2). For comparison of model fitting plots, please refer to Supplementary Figure A.

Table 2 Comparison of basic models

| Model description | LogLik | OFV ^a^ | AIC | BIC | Shrinkage |
| --- | --- | --- | --- | --- | --- |
| 1 ^a^ _Addictive | -727.81 | 1455.62 | 1465.62 | 1483.81 | 9.70% |
| 1 ^a^ _Multiplicative | -701.02 | 1402.04 | 1412.04 | 1430.23 | 13.91% |
| 1 ^a^ _Add_Multiplicative | -699.91 | 1399.82 | 1411.82 | 1433.65 | 13.43% |
| 2 ^b^ _Addictive | -727.86 | 1455.73 | 1473.73 | 1506.47 | 9.88% |
| 2 ^b^ _Multiplicative | -700.23 | 1400.45 | 1418.45 | 1451.20 | 13.38% |
| 2 ^b^ _Add_Multiplicative | -693.35 | 1386.71 | 1406.71 | 1443.09 | 13.21% |

^a^ First-order compartment model; ^b^ Two compartment model; OFV: objective function value; AIC: Akaike Information Criterion; BIC: Bayesian information criterion.

After screening all 21 covariates, it was found that Cvrc was significantly negatively correlated with CL; PLT was significantly positively correlated with CL, and RBC was significantly negatively correlated with CL (Table A). The final equation obtained by incorporating relevant variables was Ka=3.09; V/F=1635.34, CL/F=5.73 (Table 3).

**The formulas for V and CL were as follows:**

V=1635.34 * (1+(C_VRC_-1.70) * (-0.07)) * exp(nV))

Cl=5.73 * (RBC/3.49) * ^(-1.34)^ * (PLT/181.24) * ^0.46^ *(1+(C_VRC_-1.70) * (-0.12)) exp(nCL))

CMultStdev=0.36

Table 3 Final model parameters

| Parameter | Estimate | Units | Stderr | CV% | 2.5% CI | 97.5%CI |
| --- | --- | --- | --- | --- | --- | --- |
| Ka(Fix) | 3.09 | 1/h | 0.00 | 0.000 | 3.09 | 3.09 |
| V/F | 1635.34 | L | 0.17 | 10.51 | 1.30 | 1.97 |
| CL/F | 5.73 | L/h | 0.001 | 9.05 | 0.005 | 0.007 |
| tvCMultStdev | 0.36 |  | 0.02 | 6.11 | 0.32 | 0.41 |
| dCldRBC | -1.34 |  | 0.29 | -21.68 | -1.92 | -0.77 |
| dCldPLT | 0.46 |  | 0.20 | 43.24 | 0.07 | 0.85 |
| dCldCv_R_c | -0.12 |  | 0.02 | -17.19 | -0.16 | -0.08 |
| dVdC_VRC_ | -0.07 |  | 0.02 | -29.397 | -0.11 | -0.03 |
| stdev0 | 0.04 |  | 0.007 | 16.21 | 0.03 | 0.05 |

CV%: coefficient of variation; dCLdRBC, dCLdPLT and dCLC_VRC_: Effect of RBC, PLT and C_VRC_ on CL, respectively; dVdC_VRC_ ,Effect of C_VRC_ on V.

**3.3 Validation of the population pharmacokinetic model**

**3.3.1 Bootstrap Validation and Model Fit Plots**

The accuracy of the final model was examined by bootstrap validation (simulation 1500 times), and it was found that key data such as PK parameters were within a reasonable range (Table 4). In addition, the final model fitting graph had good convergence (Figure 2).

Table 4 Comparison of parameter estimates in the final model and bootstrap

| Final model results | |  | Bootstrap results | | | | |
| --- | --- | --- | --- | --- | --- | --- | --- |
| Parameter | Estimate | Mean | SD | CV% | Median | 2.50% | 97.50% |
| Ka | 3.09 | 3.09 | / | / | 3.09 | 3.09 | 3.09 |
| V/F | 1635.34 | 1636.20 | 179.53 | 10.97 | 1626.24 | 1313.12 | 2013.20 |
| CL/F | 5.73 | 5.76 | 0.60 | 10.48 | 5.74 | 4.65 | 7.07 |
| tvCMultStdev | 0.36 | 0.36 | 0.02 | 6.27 | 0.36 | 0.31 | 0.40 |
| *Θ_RBC-CL_* | -1.34 | -1.42 | 0.42 | -29.52 | -1.39 | -2.27 | -0.63 |
| *Θ_PLT-CL_* | 0.46 | 0.47 | 0.28 | 59.72 | 0.44 | 0.003 | 1.13 |
| *Θ_Cvrc-CL_* | -0.12 | -0.12 | 0.04 | -30.60 | -0.12 | -0.20 | -0.38 |
| *Θ_Cvrc-V_* | -0.07 | -0.07 | 0.04 | -33.38 | -0.07 | -0.10 | -0.02 |
| *ω*^2^_V_ | 0.091 |  |  | / | 0.091 |  |  |
| *ω*^2^ _CL_ | 0.039 |  |  | / | 0.063 |  |  |
| σ | 0.04 | 0.04 | 0.03 | / | 0.04 | 0.040 | 0.044 |

CV%: coefficient of variation; Θ_RBC-CL_: exponent for RBC as a covariate for CL: Θ_PLT-CL_: exponent for PLT, as a covariate for CL; Θ_Cvrc-CL_: exponent for C_vrc_, as a covariate for CL; Θ_Cvrc-V_: exponent for C_vrc_, as a covariate for V ω: inter-individual variation; σ: intraindividual variation; /, not applicable.

**3.3.2** **Visual forecast checking**

The VPC method was used for 1500 simulation simulations to verify the final model. The VPC diagnosis figures are shown in Figure 3. As can be seen from the figure, the 5th, 50th, and 95th quantiles of all the observed values fell within the 90%CI of the corresponding predicted values, indicating that the predicted values were in high agreement with the observed values, indicating that the prediction performance of the model was good.

**4.Discussion**

In this study, we developed a single-compartment model of oral first-level elimination and described a PPK model of nvasive fungal infections (IFIs) in renal transplant recipients treated with tacrolimus plus voriconazole. Specifically, a median tacrolimus concentration of 5.80 [4.20, 8.65] ng/mL was observed, which is consistent with the European Consensus Conference recommendations^16^. These guidelines recommend maintaining tacrolimus (FK506) whole blood concentrations between 5 and 10 ng/mL during the first 12 months after transplantation. Furthermore, the median voriconazole concentration was 0.95 [0.00, 2.64] ng/mL, which was notably higher than the median concentration of 0.00 [0.00, 0.50] ng/mL reported in a retrospective study of renal transplant recipients 15 days post-surgery^17^. This difference may be attributed to variations in dosing strategies between long-term transplant recipients and those in the early. This study found that voriconazole concentration was negatively correlated with tacrolimus clearance, RBC level was negatively correlated with clearance, and PLT level was positively correlated with clearance. Because voriconazole significantly reduced the metabolic rate of tacrolimus by inhibiting the CYP3A4 enzyme system, resulting in a decrease in its clearance. At the same time, the effect of voriconazole on liver and kidney function can indirectly affect the distribution of tacrolimus^18, 19^. Studies have found that Cvrc is significantly negatively correlated with V; Similarly, Staats et al. The interaction between voriconazole and tacrolimus at the level of drug transporters such as P-glycoprotein (P-gp) can further influence the distribution of tacrolimus^20^.^21^ Additionally, changes in protein binding due to coadministration of voriconazole may lead to changes in tacrolimus distribution^21^.

Low red blood cell levels may reflect potential liver dysfunction or other systemic problems, thereby reducing the metabolism of tacrolimus^22^. Instead, higher platelet levels may reflect certain body states (such as inflammation or other stimuli) that may promote the metabolism of tacrolimus through complex physiological mechanisms (such as increased blood flow to the liver), thereby increasing its clearance. These findings are consistent with previous studies.

The high temperature and humidity during the plum rain season create an ideal environment for the growth and dissemination of pathogenic fungi, particularly Aspergillus species^23^, leading to a significant increase in the incidence of invasive fungal infections (IFIs) among kidney transplant patients. This study specifically focuses on kidney transplant recipients suffering from invasive fungal infections. As a typical plum rain region, Changsha, Hunan, further elevating the infection risk for immunosuppressed patients. Therefore, kidney transplant recipients should adopt preventive measures such as improved air quality management, early screening, and prophylactic antifungal therapy to reduce infection rates and mortality. This phenomenon demonstrates strong regional and seasonal characteristics, warranting clinical attention. In addition, we previously conducted a retrospective study on the combined use of medications in renal transplant patients 15 days post-surgery, which provided valuable insights for subsequent research^17^. Compared with the previous study, the present study adopts a prospective design, focusing on patients one year or more after transplantation, with an emphasis on the long-term effects in this population. The sample size has also increased significantly, from 19 participants in the earlier study to 51 in this one, with a similarly high proportion of male participants (78.9% and 72.5%, respectively). Furthermore, the methods of blood sampling differed between the two studies. The current study utilized random sampling, encompassing the entire phase of drug absorption and elimination, which allowed for real-time measurement of tacrolimus and voriconazole blood concentrations. This approach minimizes the risk of data lag and enhances the accuracy of drug concentration assessments. The findings revealed significant differences in drug concentrations at the two time points. Specifically, the median tacrolimus concentration in patients one-year post-surgery was lower than in those within 15 days post-surgery (5.80 ng/mL vs. 7.90 ng/mL, respectively), whereas voriconazole concentrations were higher. Moreover, in both studies, a significant effect of voriconazole concentration on the volume of distribution and clearance of tacrolimus was observed. These results provide critical insights into the pharmacokinetics of these drugs in long-term renal transplant recipients and underscore the importance of individualized treatment strategies for this population.

At the same time, the study unexpectedly found that for patients taking tacrolimus for a long time, clinicians often gave smaller doses. This may be due to a decrease in the target concentration range of tacrolimus as time after transplantation increases.

There are some limitations. This study utilized the immediate-release (IR) formulation of tacrolimus, which is typically dosed twice daily. Due to the differences in pharmacokinetics, the findings of this study may not be directly applicable to other formulations of tacrolimus, such as prolonged-release or extended-release formulations. The limitation of sample size may affect the generalizability of the study conclusions. Although this study revealed a significant effect of voriconazole on the pharmacokinetics of tacrolimus, the small sample size may limit the generalizability of the study results in different populations. Therefore, a larger multicenter study should be conducted in the future to expand the sample size, further verify the conclusions of this study, and improve the statistical power and the wide applicability of the conclusions. At present, we have begun to prepare for a multicenter study. The study of group behavior and drug interaction in this study mainly focused on clinical observation data, and the exploration of its potential mechanisms was insufficient. Group behavior is inherently dynamic and complex and is affected by multiple factors. Drug interactions often produce different reactions with changes in physiological state. Future studies should combine longer longitudinal tracking data to explore drug metabolic pathways under different environments and conditions and reveal the multidimensional mechanisms of drug interactions. In summary, the limitations of this study provide guidance for future multicenter, large sample, and interdisciplinary in-depth research, aiming to optimize medication strategies and improve clinical treatment effects through more comprehensive data and theoretical support.

**5. Conclusions**

This study successfully constructed a population pharmacokinetic model for renal transplant patients taking tacrolimus and voriconazole simultaneously. The model had good predictive ability and provided valuable insights to clinicians to help optimizing tacrolimus dosing.

**6. Conflict of Interest**

No conflict of interest

**7. Author contributions**

Conceptualization, Miao Yan, Bikui Zhang. and Zhihua Sun; Methodology, Zhihua Sun and Yichang Zhao; Software, Zhihua Sun and Yichang Zhao; Validation, Zhihua Sun and Yichang Zhao.; Formal analysis, Zhihua Sun; Investigation, Zhihua Sun, Huailin Cai , Fenghua Peng ; Resources, Bikui Zhang; Data management, Zhihua Sun; Writing—original draft preparation, Zhihua Sun, .; Writing-review and editing, Zhihua Sun, Huaiyuan Liu, and Jiakai Li. Visualization, Zhihua Sun; Supervision, Zhihua Sun Feng Yu and Yichang Zhao; Project administration, Feng Yu; Funding acquisition, Miao Yan. All authors have read and agreed to the published version of the manuscript.

**8. Funding**

This research was funded by Hunan Medical Association with the founding number of [HMA202001002]; This research was funded by the Interna-tional Research Center for Precision Medicine, Transformative Technology, and Software Services, Hunan, China; This research was funded by Research Project established by Chinese Pharmaceutical Association Hospital Phamacy department [NO. CPA-Z05-ZC-2024002]; This research was funded by Hunan Provincial Health High-Level Talent Scientific Research[R2023061].

**9. Acknowledgements**

Thanks to all participants.

**10. Informed Consent Statement**

Written informed consent was obtained from the patient for publication of this case report and accompanying images. A copy of the written consent is available for review by the Editor-in-Chief of this journal upon request

**11.Figure**


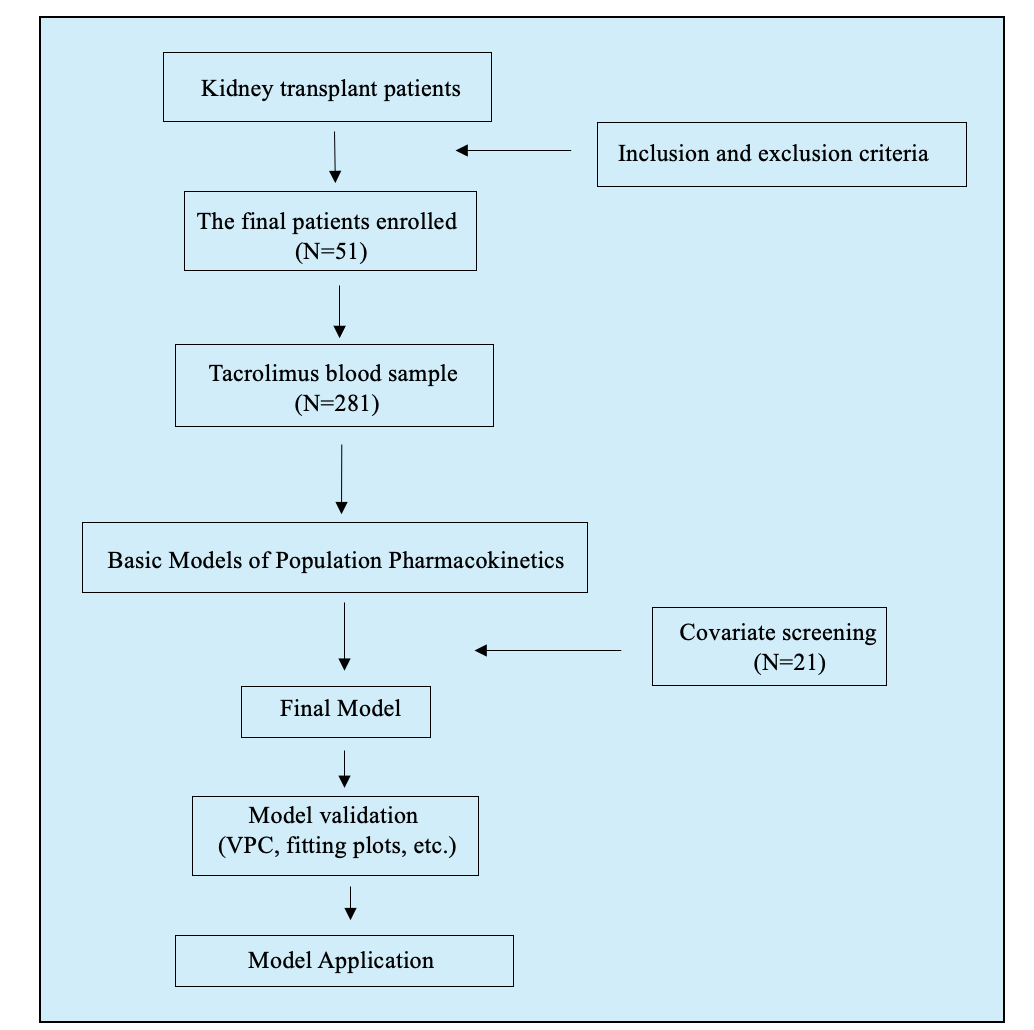


Figure 1 Research flow chart


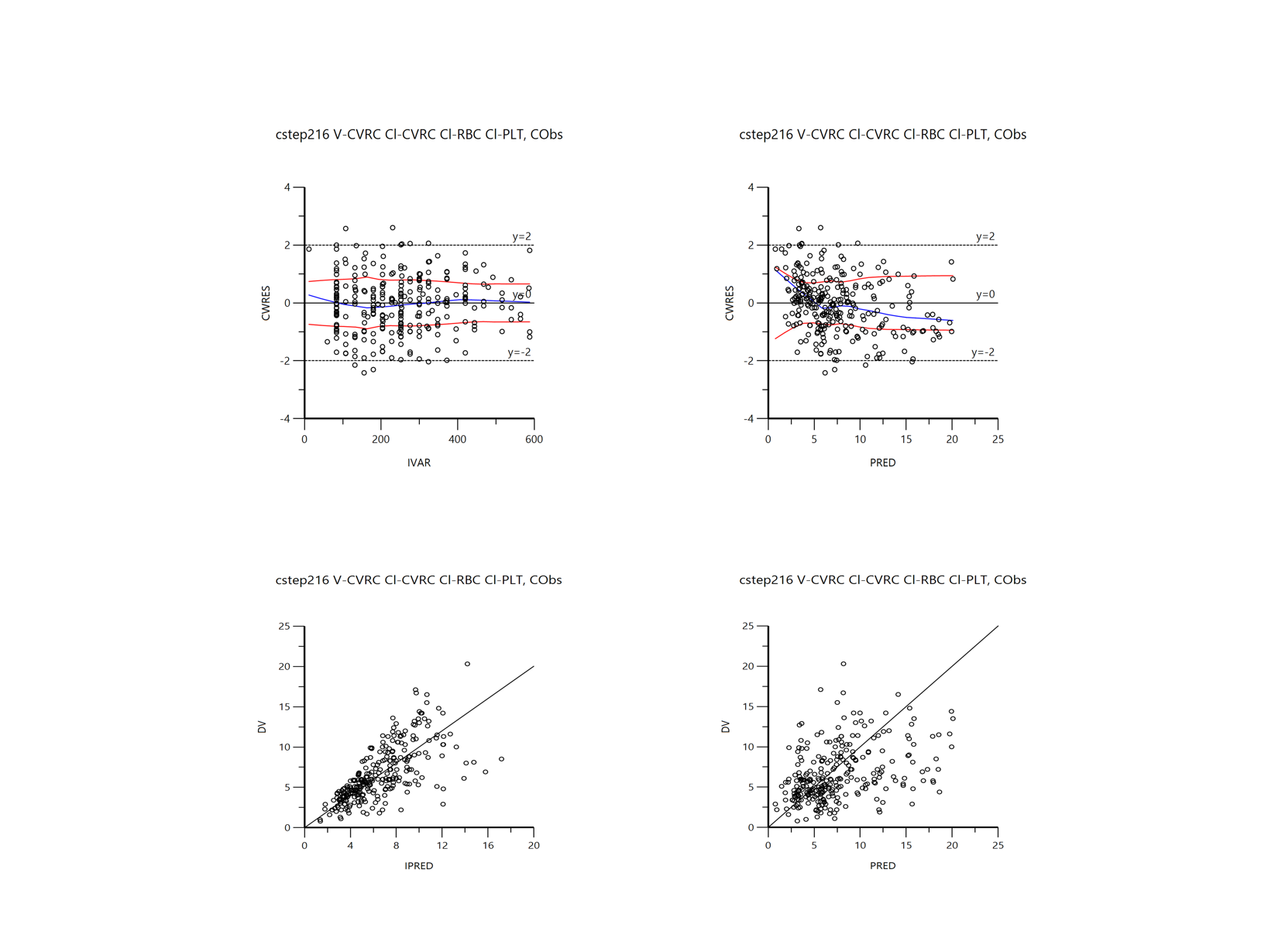


Figure 2 Final model fit plot


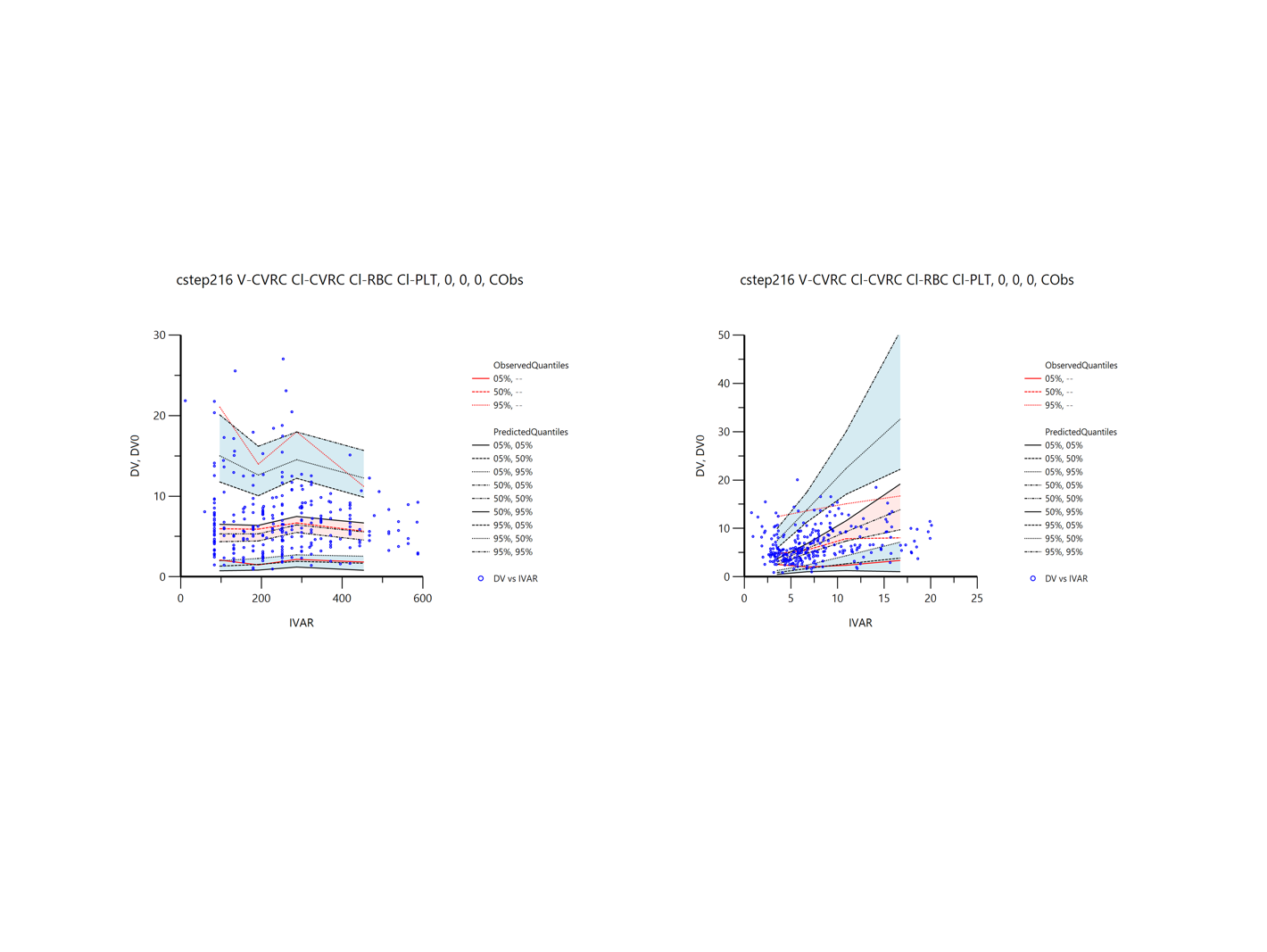


Figure 3 VPC simulation diagram

**12. References**

(1) Augustine, J. Kidney transplant: New opportunities and challenges. *Cleve Clin J Med* **2018**, *85* (2), 138-144. DOI: 10.3949/ccjm.85gr.18001 From NLM.

(2) Voora, S.; Adey, D. B. Management of Kidney Transplant Recipients by General Nephrologists: Core Curriculum 2019. *Am J Kidney Dis* **2019**, *73* (6), 866-879. DOI: 10.1053/j.ajkd.2019.01.031 From NLM.

(3) Gong, F.; Hu, H.; Ouyang, Y.; Liao, Z. Z.; Kong, Y.; Hu, J. F.; He, H.; Zhou, Y. Physiologically-based pharmacokinetic modeling-guided rational combination of tacrolimus and voriconazole in patients with different CYP3A5 and CYP2C19 alleles. *Toxicol Appl Pharmacol* **2023**, *466*, 116475. DOI: 10.1016/j.taap.2023.116475 From NLM.

(4) Maertens, J. A.; Raad, II; Marr, K. A.; Patterson, T. F.; Kontoyiannis, D. P.; Cornely, O. A.; Bow, E. J.; Rahav, G.; Neofytos, D.; Aoun, M.; et al. Isavuconazole versus voriconazole for primary treatment of invasive mould disease caused by Aspergillus and other filamentous fungi (SECURE): a phase 3, randomised-controlled, non-inferiority trial. *Lancet* **2016**, *387* (10020), 760-769. DOI: 10.1016/s0140-6736(15)01159-9 From NLM.

(5) Coto, E.; Tavira, B.; Suárez-Álvarez, B.; López-Larrea, C.; Díaz-Corte, C.; Ortega, F.; Alvarez, V. Pharmacogenetics of tacrolimus: ready for clinical translation? *Kidney Int Suppl (2011)* **2011**, *1* (2), 58-62. DOI: 10.1038/kisup.2011.14 From NLM.

(6) Choi, Y.; Jiang, F.; An, H.; Park, H. J.; Choi, J. H.; Lee, H. A pharmacogenomic study on the pharmacokinetics of tacrolimus in healthy subjects using the DMETTM Plus platform. *Pharmacogenomics J* **2017**, *17* (1), 105-106. DOI: 10.1038/tpj.2016.85 From NLM.

(7) Staatz, C. E.; Goodman, L. K.; Tett, S. E. Effect of CYP3A and ABCB1 single nucleotide polymorphisms on the pharmacokinetics and pharmacodynamics of calcineurin inhibitors: Part I. *Clin Pharmacokinet* **2010**, *49* (3), 141-175. DOI: 10.2165/11317350-000000000-00000 From NLM.

(8) Jeong, S.; Nguyen, P. D.; Desta, Z. Comprehensive in vitro analysis of voriconazole inhibition of eight cytochrome P450 (CYP) enzymes: major effect on CYPs 2B6, 2C9, 2C19, and 3A. *Antimicrob Agents Chemother* **2009**, *53* (2), 541-551. DOI: 10.1128/aac.01123-08 From NLM.

(9) Kluwe, F.; Michelet, R.; Huisinga, W.; Zeitlinger, M.; Mikus, G.; Kloft, C. Towards Model-Informed Precision Dosing of Voriconazole: Challenging Published Voriconazole Nonlinear Mixed-Effects Models with Real-World Clinical Data. *Clin Pharmacokinet* **2023**, *62* (10), 1461-1477. DOI: 10.1007/s40262-023-01274-y From NLM.

(10) Lloberas, N.; Grinyó, J. M.; Colom, H.; Vidal-Alabró, A.; Fontova, P.; Rigo-Bonnin, R.; Padró, A.; Bestard, O.; Melilli, E.; Montero, N.; et al. A prospective controlled, randomized clinical trial of kidney transplant recipients developed personalized tacrolimus dosing using model-based Bayesian Prediction. *Kidney Int* **2023**, *104* (4), 840-850. DOI: 10.1016/j.kint.2023.06.021 From NLM.

(11) Vanhove, T.; Bouwsma, H.; Hilbrands, L.; Swen, J. J.; Spriet, I.; Annaert, P.; Vanaudenaerde, B.; Verleden, G.; Vos, R.; Kuypers, D. R. J. Determinants of the Magnitude of Interaction Between Tacrolimus and Voriconazole/Posaconazole in Solid Organ Recipients. *Am J Transplant* **2017**, *17* (9), 2372-2380. DOI: 10.1111/ajt.14232 From NLM.

(12) Ota, R.; Hirata, A.; Noto, K.; Yokoyama, S.; Hosomi, K.; Takada, M.; Matsuoka, H. Relationship between the blood concentrations of tacrolimus and voriconazole in hematopoietic stem cell transplant recipients . *Int J Clin Pharmacol Ther* **2019**, *57* (11), 561-566. DOI: 10.5414/cp203539 From NLM.

(13) Chen, W.; Wang, X.; Li, B.; Qin, W.; Li, S.; Wang, X.; Chen, W.; Zhang, X.; Li, P.; Zuo, X. Effects of Voriconazole Exposure on the Pharmacokinetics of Tacrolimus in Lung Transplantation Patients, Based on Therapeutic Drug Monitoring Data. *J Clin Pharmacol* **2022**, *62* (10), 1310-1320. DOI: 10.1002/jcph.2066 From NLM.

(14) Burrows, F. S.; Carlos, L. M.; Stojanova, J.; Marriott, D. J. E. It cuts both ways: A single-center retrospective review describing a three-way interaction between flucloxacillin, voriconazole and tacrolimus. *Int J Antimicrob Agents* **2023**, *62* (3), 106908. DOI: 10.1016/j.ijantimicag.2023.106908 From NLM.

(15) Laboratories, A. ARCHITECT Tacrolimus Reagent Kit Instructions for IL77-35. **2009**.

(16) Wallemacq, P.; Armstrong, V. W.; Brunet, M.; Haufroid, V.; Holt, D. W.; Johnston, A.; Kuypers, D.; Le Meur, Y.; Marquet, P.; Oellerich, M.; et al. Opportunities to optimize tacrolimus therapy in solid organ transplantation: report of the European consensus conference. *Ther Drug Monit* **2009**, *31* (2), 139-152. DOI: 10.1097/FTD.0b013e318198d092 From NLM.

(17) Zhao, Y. C.; Sun, Z. H.; Li, J. K.; Liu, H. Y.; Zhang, B. K.; Xie, X. B.; Fang, C. H.; Sandaradura, I.; Peng, F. H.; Yan, M. Individualized dosing parameters for tacrolimus in the presence of voriconazole: a real-world PopPK study. *Front Pharmacol* **2024**, *15*, 1439232. DOI: 10.3389/fphar.2024.1439232 From NLM.

(18) Theuretzbacher, U.; Ihle, F.; Derendorf, H. Pharmacokinetic/pharmacodynamic profile of voriconazole. *Clin Pharmacokinet* **2006**, *45* (7), 649-663. DOI: 10.2165/00003088-200645070-00002 From NLM.

(19) Neofytos, D.; Lombardi, L. R.; Shields, R. K.; Ostrander, D.; Warren, L.; Nguyen, M. H.; Thompson, C. B.; Marr, K. A. Administration of voriconazole in patients with renal dysfunction. *Clin Infect Dis* **2012**, *54* (7), 913-921. DOI: 10.1093/cid/cir969 From NLM.

(20) Fu, R.; Tajima, S.; Suetsugu, K.; Watanabe, H.; Egashira, N.; Masuda, S. Biomarkers for individualized dosage adjustments in immunosuppressive therapy using calcineurin inhibitors after organ transplantation. *Acta Pharmacol Sin* **2019**, *40* (2), 151-159. DOI: 10.1038/s41401-018-0070-2 From NLM.

(21) Yuan, Z. Q.; Qiao, C.; Yang, Z. C.; Yu, L.; Sun, L. N.; Qian, Y.; Zhang, X. H.; Meng, L.; Zhang, X. Y.; Wang, Y. Q. The Impact of Plasma Protein Binding Characteristics and Unbound Concentration of Voriconazole on Its Adverse Drug Reactions. *Front Pharmacol* **2020**, *11*, 505. DOI: 10.3389/fphar.2020.00505 From NLM.

(22) Venkataramanan, R.; Swaminathan, A.; Prasad, T.; Jain, A.; Zuckerman, S.; Warty, V.; McMichael, J.; Lever, J.; Burckart, G.; Starzl, T. Clinical pharmacokinetics of tacrolimus. *Clin Pharmacokinet* **1995**, *29* (6), 404-430. DOI: 10.2165/00003088-199529060-00003 From NLM.

(23) Valencia-Quintana, R.; Milić, M.; Jakšić, D.; Šegvić Klarić, M.; Tenorio-Arvide, M. G.; Pérez-Flores, G. A.; Bonassi, S.; Sánchez-Alarcón, J. Environment Changes, Aflatoxins, and Health Issues, a Review. *Int J Environ Res Public Health* **2020**, *17* (21). DOI: 10.3390/ijerph17217850 From NLM.
